# A Split NanoLuc Reporter Quantitatively Measures Circular RNA IRES Translation

**DOI:** 10.3390/genes13020357

**Published:** 2022-02-16

**Authors:** Priyanka Sehta, Ann-Marie Wilhelm, Shu-Jun Lin, Michelle A. Urman, Haley A. MacNeil, Gabriele Fuchs

**Affiliations:** 1 Department of Biological Sciences, University at Albany, SUNY, 1400 Washington Ave, Albany, NY 12222, USA; psehta@albany.edu (P.S.); awilhelm@albany.edu (A.-M.W.); slinchin@albany.edu (S.-J.L.); murman@albany.edu (M.A.U.); hmacneil@albany.edu (H.A.M.); 2 The RNA Institute, 1400 Washington Ave, Albany, NY 12222, USA

**Keywords:** internal ribosome entry site, RNA circles, split NanoLuc, reporter assay

## Abstract

Internal ribosomal entry sites (IRESs) are RNA secondary structures that mediate translation independent from the m7G RNA cap. The dicistronic luciferase assay is the most frequently used method to measure IRES-mediated translation. While this assay is quantitative, it requires numerous controls and can be time-consuming. Circular RNAs generated by splinted ligation have been shown to also accurately report on IRES-mediated translation, however suffer from low yield and other challenges. More recently, cellular sequences were shown to facilitate RNA circle formation through backsplicing. Here, we used a previously published backsplicing circular RNA split GFP reporter to create a highly sensitive and quantitative split nanoluciferase (NanoLuc) reporter. We show that NanoLuc expression requires backsplicing and correct orientation of a bona fide IRES. In response to cell stress, IRES-directed NanoLuc expression remained stable or increased while a capped control reporter decreased in translation. In addition, we detected NanoLuc expression from putative cellular IRESs and the Zika virus 5′ untranslated region that is proposed to harbor IRES function. These data together show that our IRES reporter construct can be used to verify, identify and quantify the ability of sequences to mediate IRES-translation within a circular RNA.

## 1. Introduction

All eukaryotic mRNAs are co-transcriptionally capped with a m7G cap [1]. This cap structure is not only required to protect mRNAs from exonucleases and degradation, but also to enable translation initiation. In the cytoplasm, the m7G cap is bound by the cap-binding protein eukaryotic initiation factor (eIF) 4E, which binds to the scaffolding protein eIF4G [2,3]. Together, these two proteins along with the helicase eIF4A, form the initiation complex eIF4F, a key complex for translation initiation [4,5]. The 40S ribosomal subunit is recruited to the mRNA through its interaction with the eIF3 complex [2,3]. Many viruses initiate translation independent of the eIF4F complex. Instead, these viruses use RNA secondary structures known as internal ribosomal entry sites (IRESs) to recruit ribosomes to the viral RNA [6,7]. IRESs were initially discovered in poliovirus and encephalomyocarditis virus (EMCV) but were since found in a variety of viruses from different virus families [8,9,10].

Viruses employ specific strategies to take over the translation machinery in cells. Poliovirus and other viruses from the Picornaviridae family express viral proteases that cleave translation initiation factors eIF4G and polyA-binding protein (PABP), which prevents the translation of the majority of mRNAs [11,12,13]. However, translation of the poliovirus genome can continue as translation of the viral genome is initiated via their IRES and does not require all translation initiation factors [14]. Interestingly, select mRNAs continue to be translated under those conditions, and it was speculated that certain cellular mRNAs may also have an IRES [15,16].

Because cellular mRNAs are always capped, the question of whether cellular mRNAs can have an IRES was heavily debated [17]. Specifically, the findings that certain mRNAs were translated during poliovirus infection when cellular translation was mostly inactive provided strong evidence for IRES structures on cellular mRNAs [15,16]. In similar experiments using chemical stressors, translation of certain mRNAs can also be observed [18]. These mRNAs that can be translated during conditions of translational shut-off are frequently mRNAs that regulate cell survival and apoptosis—functions critical for cells undergoing stress [19]. Cellular RNAs that may contain an IRES include c-Myc, endoplasmatic reticulum chaperone BiP and eIF4G2, which is also known as DAP5 [19,20].

To determine if an RNA contains an IRES, a dicistronic luciferase assay is typically employed [21]. Such plasmid encodes two open reading frames (ORFs), most frequently *Renilla* and firefly luciferases, in a sequential orientation with an IRES or a putative IRES in between the ORFs. Following transcription of this plasmid, the *Renilla* luciferase in the first cistron is translated via the canonical cap-dependent translation initiation pathway. In contrast, the firefly luciferase ORF is translated via an IRES. Although this assay is straightforward, great care must be taken to rule out false-positive results due to the presence of cryptic promoters, cryptic splice sites, and by ribosome readthrough [21]. Cryptic promoters can be ruled out by RNA transfection, and qRT-PCR where equal ratios of both cistrons should be detected. Cryptic splice sites can be detected by northern blotting and qRT-PCR, while ribosome readthrough can be prevented through the insertion of a hairpin structure downstream of the stop codon of the first cistron and upstream of the proposed IRES. These caveats have prompted many to look for alternative assays to quantify and identify IRES sequences.

RNA circularization has been proposed and shown to be an alternate more accurate approach to test for IRES activity [22]. Circular RNAs can be generated by a variety of chemical and enzymatic methods [23] and contain neither the m7G cap nor a poly-A tail. They cannot be translated via the cap-dependent translation initiation pathway, however require an IRES for ribosome recruitment and translation. One commonly used way to create circular RNAs is by splinted ligation, where a splint oligo anneals to both RNA ends to bring them in close proximity for ligation. Unfortunately, splinted ligations are frequently inefficient, making this technique quite challenging. Recent research has revealed that cells can also create circular RNAs in a process called backsplicing [24,25]. During backsplicing, a downstream splice donor site is spliced to an upstream splice acceptor site, which releases a circular RNA. The linear RNA leftover after backsplicing may be spliced and translated into a protein via cap-dependent translation or degraded. Cellular RNA circles have been proposed to function as a sponge for microRNAs or proteins [26]. IRES-containing circular RNA could also be translated to yield novel proteins or peptides that would not be generated through the translation of the linear RNA.

It was shown that the full-length intronic repeats of the ZKSCAN1 gene can mediate RNA circularization [27,28]. They created a mammalian circle splice reporter with a split GFP ORF that, upon backsplicing, would be fused. GFP translation was successfully mediated via the inclusion of the EMCV IRES, while the inclusion of this IRES in the reverse orientation did not facilitate GFP production [27]. Although GFP reporters are very commonly used, they have some disadvantages. First, due to cellular autofluorescence, low levels of GFP expression might be masked or barely detectable above the background from autofluorescence. Second, quantitative measurements of fluorescence are typically done by fluorescence-activated cell sorting (FACS), which requires special training on a FACS instrument. In contrast to fluorescence-based measurements, the background from luminescence is extremely low. Further, luminescence has a large linear dynamic range that spans five orders of magnitude [29]. Lastly, luminometers are rather cheap instruments that do not require extensive training and expertise. Taken together, a luminescent-based split ORF reporter could be a valuable reporter to measure circular RNA and IRES-mediated translation. Based on the split GFP plasmid by Kramer et al., we developed a simple and quantitative assay to measure IRES-mediated translation from a circular RNA construct via luminescence. We replaced the split GFP gene in the original ZKSCAN construct by Kramer et al. with a split NanoLuc ORF to produce a new reporter construct. Using this new construct, we showed that backsplicing and an IRES are required for NanoLuc production. Further, the joint expression of the two NanoLuc fragments is unable to complement, resulting in very low background measurements. Following the insertion of multiple putative IRES sequences, we confirmed several cellular IRESs and identified novel IRESs. We lastly converted the plasmid into a dual-luciferase reporter, where firefly luciferase measures cap-dependent translation, while the NanoLuc reports on IRES-mediated translation. Through this approach, issues of the conventional dual-luciferase reporter assay can be avoided, increasing confidence in the observed results and providing an easy-to-use quantitative IRES reporter assay.

## 2. Materials and Methods

### 2.1. Cloning

A backsplicing reporter plasmid pcDNA3.1(+) ZKSCAN1 MCS-WT Split GFP [27] was purchased as a bacterial agar stab from Addgene (#69908). The split NanoLuc gene block EMCV split NanoLuc 117_118 was purchased from Integrated DNA Technologies (IDT DNA). The gene block template was PCR-amplified with primers ZKSCAN Fwd and ZKSCAN Rev (see Figure S1) using Taq DNA polymerase (New England Biolabs, NEB), while the ZKSCAN plasmid backbone was amplified with the reverse complement primers and Q5 DNA polymerase (NEB), according to the manufacturer’s protocol. The plasmid and gene block were assembled using the HiFi Assembly kit (NEB). The plasmid DNA template was removed using the DpnI restriction enzyme and transformed into DH5alpha cells and grown on LB-Carbenicillin plates. Following miniprep plasmid isolation, the correct construct was confirmed by Sanger sequencing.

For the EMCV reverse IRES control and the Myc, DAP5 and c-Jun sequences, sequences were PCR-amplified using Q5 DNA polymerase (see Table S1 for all primers). Following restriction digest with SbfI and EcoRV, the inserts were ligated into the ZKSCAN split NanoLuc plasmid digested with the same restriction enzymes.

For the insertion of a short 9 bp unstructured linker sequence, two overlapping oligonucleotides with SbfI and EcoRV overhangs were phosphorylated with T4 polynucleotide kinase (NEB) using DNA ligase buffer (NEB), annealed and inserted into the ZKSCAN split NanoLuc plasmid.

The N- and C-terminal halves were individually amplified from the ZKSCAN plasmid and inserted into the HindIII and BamHI restriction sites of pcDNA3.

Ligations were performed using T4 DNA Ligase (NEB) following the manufacturer’s instructions.

For the donor and the acceptor splice site mutants, PCR and KLD treatment were performed, as described in the Q5 mutagenesis kit (NEB).

For insertion of the ZIKV UTRs, two gene blocks containing the ZIKV 5′-3′ UTRs and 3′-5′ UTRs were purchased from GENEWIZ/Azenta Life Sciences. These sequences and the single 5′ UTR and the 3′ UTR were PCR amplified and ligated into the SbfI and EcoRV sites of the ZKSCAN split NanoLuc plasmid.

To generate the dual-luciferase construct, the firefly luciferase was amplified from pGL4.13 plasmid and inserted into the NheI and BamHI sites of the linker and the c-Myc IRES-containing ZKSCAN NanoLuc plasmids.

All constructs were transformed into DH5α cells and grown overnight on LB-Carbenicillin plates. Following miniprep plasmid isolation, the correct construct was confirmed by Sanger sequencing.

### 2.2. Cell Culture

Adherent HeLa S3 cells were grown in Dulbecco’s modified Eagle’s medium (SigmaAldrich, St. Louis, MO, USA) supplemented with 10% (*v/v*) FBS (Avantor-Seradigm, Radnor, PA, USA), 2 mM L-glutamine (ThermoFisher, Waltham, MA, USA).

### 2.3. Luciferase Assay

An amount of 20,000 Hela cells were seeded per well of a 96-well plate. The next day, cells were co-transfected with a mixture of 90 ng ZKSCAN split NanoLuc plasmid and 10 ng pGL4.13 firefly luciferase plasmid or 100 ng of the dual-luciferase containing plasmid using Lipofectamine 3000 (ThermoFisher), following the manufacturer’s protocol. Cells were grown for 24 h, then lysed in passive lysis buffer (Promega, Madison, WI, USA). Firefly luciferase and NanoLuc activities were measured in a Glomax instrument using the Dual Nano-Glo kit (Promega).

## 3. Results

IRESs are well-known to mediate translation through a cap-independent mechanism and the dicistronic luciferase reporter system is the gold standard to measure IRES-mediated translation. However, the dual-luciferase system can yield false-positive results and hence requires several control experiments to deduce the correct data. To overcome such issues, we measured translation from a circular RNA generated from a new backsplicing reporter construct as an alternative approach. While generating circular RNAs by splinted ligation suffers from low yield, recent research has shown that cells can synthesize RNA circles by a backsplicing event that is facilitated by complementary sequences upstream and downstream of the RNA sequence of interest included in the circle. In this study, we used a previously published plasmid (pcDNA3.1(+) ZKSCAN1 MCS-WT Split GFP) to develop a quantitative circular RNA reporter [27]. Instead of a split GFP ORF, we inserted a split NanoLuc ORF to allow for quantitative measurements of IRES-mediated translation by luminescence (pcDNA3.1(+) ZKSCAN1 split NanoLuc). NanoLuc was utilized as it is 100 times more sensitive than firefly luciferase [30] and can compensate for the low frequency of backsplicing events.

### 3.1. Development of a Split NanoLuc Reporter

**Figure 1:**
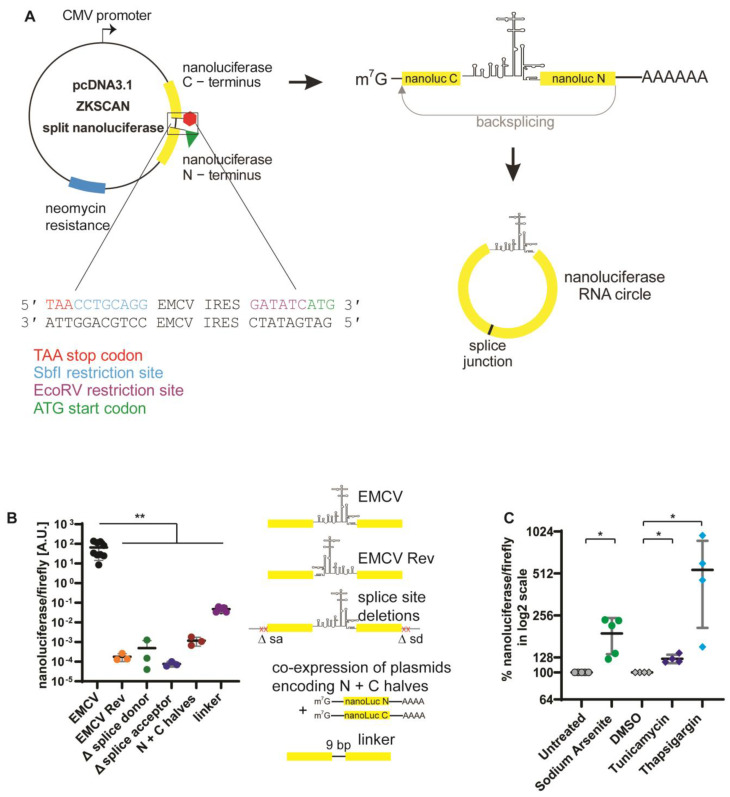
A split NanoLuc reporter quantitatively measures translation from a circular RNA. (**A**) ZKSCAN split NanoLuc plasmid construct contains SbfI and EcoRV restriction sites, into which the EMCV IRES had been inserted. Upon transcription, a linear capped and polyadenylated transcript is formed. Upon backsplicing, the split NanoLuc open reading frame (ORF) is fused, which results in active NanoLuc protein. (**B**) NanoLuc over firefly luciferase luminescence ratios is plotted for the different control constructs containing the EMCV IRES in the forward (EMCV) or reverse orientation (EMCV Rev), the EMCV IRES in the forward direction with the intronic splice donor (∆ splice donor) or acceptor sites (∆ splice acceptor) deleted, co-expression of N− and C−terminal fragments (N + C halves) from two co-transfected DNA plasmids, and the empty plasmid with a short 9 bp unstructured linker inserted into the SbfI and EcoRV sites (linker). Schematics corresponding to the constructs tested here are shown to the right. (**C**) NanoLuc expression from circular RNAs in response to cellular stress induced by sodium arsenite, tunicamycin and thapsigargin compared to an untreated and a DMSO control. NanoLuc expression normalized to firefly luciferase is displayed in a percentage. All experiments were performed at least in triplicate. Error bars represent the standard deviation. Significances were calculated using an unpaired *t* test with Welch’s correction with *p* ≤ 0.05 = *, *p* ≤ 0.01 = **.

For the split NanoLuc reporter, we sought to split the NanoLuc ORF in a position where both NanoLuc fragments, when co-expressed, would not be able to yield active NanoLuc protein. Zhao et al. showed that a NanoLuc protein split between amino acid positions 117 and 118 is unable to complement and yield active NanoLuc [31]. Hence, we designed a gene block where the EMCV IRES was flanked by the C-terminal NanoLuc fragment upstream and the N-terminal NanoLuc fragment downstream. The complementary sequences from the ZKSCAN plasmid were located upstream and downstream of the NanoLuc fragments to allow for insertion of the resulting gene block into the PCR-amplified pcDNA3.1(+) ZKSCAN1 MCS-WT Split GFP plasmid via Gibson assembly cloning. These sequences also facilitate the backsplicing event that forms the RNA circle. Upon transfection of the plasmid, the CMV promoter promotes transcription to generate a linear RNA, which is capped and polyadenylated on the 5′ and 3′ ends, respectively. Upon backsplicing, the split ORF from the NanoLuc gene is fused, resulting in a circular RNA containing the EMCV IRES, followed by the entire NanoLuc ORF and an in-frame stop codon (Figure 1A).

Following co-transfection of the ZKSCAN EMCV split NanoLuc and the pGL4.13 plasmid encoding a firefly luciferase protein, both NanoLuc and firefly luciferase activities were measured and the NanoLuc over firefly ratio was plotted (Figure 1B). To show that the insertion of an IRES is required for translation, the EMCV IRES was inserted in the reverse orientation, which completely abolished NanoLuc production (Figure 1B). We further tested if backsplicing was required for NanoLuc expression by removing either the two conserved nucleotides in the intron of the splice donor or the splice acceptor site by site-directed mutagenesis. Loss of splicing was confirmed by Northern blotting (Figure S2). Removal of those conserved nucleotides indeed abolished NanoLuc expression, suggesting that backsplicing is a required step. Co-expression of the N- and C-terminal NanoLuc fragments from two co-transfected mammalian expression plasmids encoding either fragment also displayed minimal expression. Although insertion of a short, 9-nt unstructured linker increased NanoLuc expression about 15-fold to 0.047 ± 0.012 A.U, these values were 1600-fold lower than the NanoLuc expression values from the EMCV IRES. (Figure 1B). Since IRES-mediated translation increases in the presence of stressors, we next exposed cells transfected with the ZKSCAN EMCV split NanoLuc reporter for 5 h to 100 μM sodium arsenite, 20 μM tunicamycin and 20 μM thapsigargin. All these reagents activate the integrated stress response, which causes phosphorylation of the translation initiation factor eIF2α at serine 51 [32,33]. Phosphorylation of eIF2α is known to increase IRES-mediated translation. We expected that stress induction would result in increased NanoLuc and lower firefly luciferase expression, as firefly luciferase cap-dependent translation would be inhibited. Following normalization to the untreated or DMSO controls, all stress-inducing chemicals displayed an increase in the ratio compared to the control. The DMSO treatment control was used for the normalization of tunicamycin and thapsigargin-treated samples, as those two compounds are dissolved in DMSO. Tunicamycin had a moderate effect compared to both sodium arsenite and thapsigargin (Figure 1C).

### 3.2. Putative IRESs

**Figure 2:**
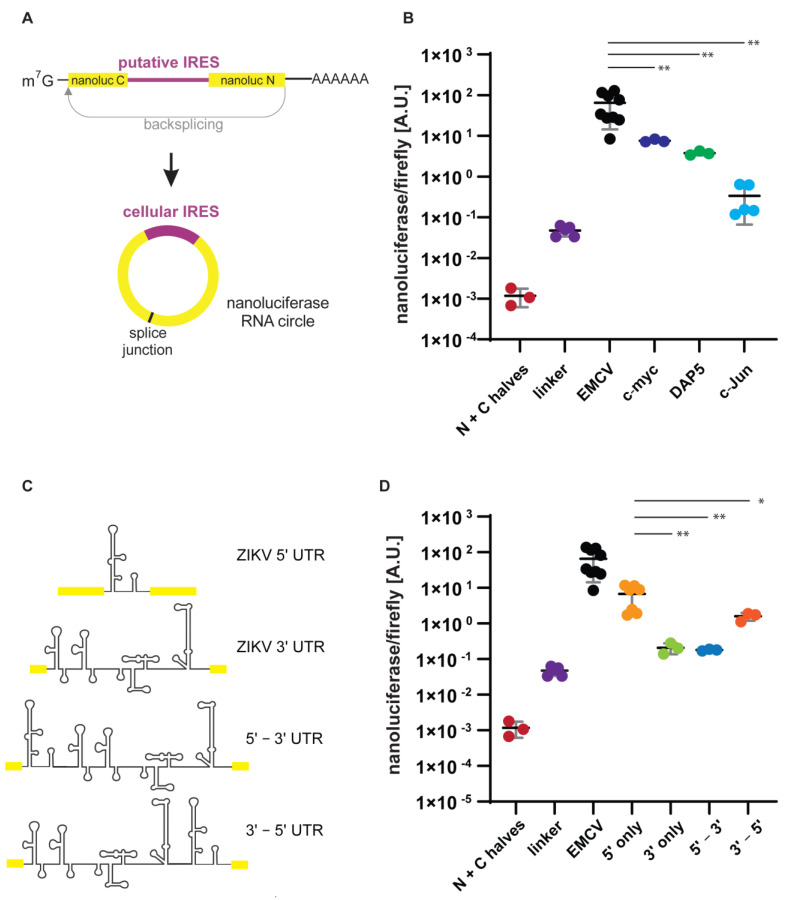
The relative activity of cellular IRESs can be quantified with a split NanoLuc reporter. (**A**) Putative cellular IRES sequences for c-Myc, DAP5 and c-Jun were cloned and inserted into the SbfI and EcoRV sites of the split NanoLuc reporter, generating a linear and a circular RNA. (**B**) Relative activity of cellular IRESs is measured by NanoLuc over firefly luciferase ratio. All experiments were performed at least in triplicate. Error bars represent the standard deviation. (**C**) ZIKV 5′ and 3′ UTRs RNA secondary structures and secondary structures in a combination of 5′-3′, 3′-5′ UTRs (**D**) NanoLuc over firefly luciferase ratio from ZIKV 5ʹ and 3ʹ UTRs alone, or in a combination of 5′-3′, 3′-5′ UTRs. Data for co-expression of N− and C−terminal fragments (N + C halves), the empty plasmid with a short 9 bp unstructured linker, and the EMCV IRES are identical to the data from Figure 1B, however, were plotted again for direct comparison. All experiments were performed at least in triplicate. Error bars represent the standard deviation. Significances were calculated using an unpaired t test with Welch’s correction with *p* ≤ 0.05 = *, *p* ≤ 0.01 = **.

The EMCV IRES is a strong viral IRES that is frequently used as a positive control for IRES-mediated translation. Since many positive-sense viral RNA genomes do not use the typical m7G cap, and these viruses often express proteins that disrupt cap-dependent translation by cleaving translation initiation factors, functional IRES sequences were easily proven. In contrast, cellular mRNAs are always capped during synthesis, which has hampered research into their ability to mediate cap-independent translation. To test various cellular IRESs’ ability to mediate translation from a circular RNA, we PCR-amplified and ligated the putative cellular IRES sequences from c-Myc, DAP5 and c-Jun into the split NanoLuc reporter (Figure 2A). IRES activity from the c-Myc and DAP5 5′ UTRs was supported through various experimental approaches [20,34,35]. In contrast, only one two-decade-old report described IRES activity within the chicken c-Jun 5′ UTR, until a more recent report described c-Jun IRES-activity in glioblastoma tumor tissue [36,37]. While the c-Myc and DAP5 5′ UTRs robustly mediated NanoLuc synthesis, NanoLuc levels from the c-Jun 5′ UTR only weakly produced NanoLuc (Figure 2B). These results are consistent with previous findings that c-Myc and DAP5 RNAs can be found in actively translated gradient fractions during poliovirus infection, where only cellular RNAs that are translated via IRES-mediated translation can be found. Interestingly, the c-Jun 5′ UTR mediates about 10 times more NanoLuc translation than the linker control (Figure 1B and Figure 2B), suggesting that the c-Jun IRES may be weak.

One of the best-studied viral IRESs is the IRES from hepatitis C virus, which is a member of the Flaviviridae family. Although members of the genus flavivirus have been shown to use an RNA cap structure, there is evidence from the Dengue virus that these viruses may also use a cap-independent pathway for translation initiation [38]. More recently, experiments with the Zikavirus (ZIKV) UTRs in the context of a dual-luciferase reporter suggest that ZIKV may utilize an IRES within its 5′ UTR for translation [39]. Using the split NanoLuc reporter, we tested the ability of the ZIKV 5′ UTR and 3′ UTR to facilitate circular RNA translation (Figure 2C). Because translation is known to bring together both 5′ and 3′ UTRs, we also tested if a combination of the two UTRs could facilitate translation. Because the UTRs would be located end-to-head in a circular RNA we inserted a gene block with the 3′ UTR upstream of the 5′ UTR and a second gene block with the 3′ UTR following the 5′ UTR (Figure 2C). Interestingly, we found that the ZIKV 5′ UTR alone, or downstream of the 3′ UTR, was indeed able to mediate NanoLuc translation from the circular RNA (Figure 2D). These results suggest that the ZIKV 5′ UTR contains an IRES, confirming results by Song et al., who previously showed in the context of a dual-luciferase reporter that the ZIKV 5′ UTR can facilitate translation [39].

### 3.3. Generation of a Single-Plasmid, Dual-Luciferase Reporter

**Figure 3:**
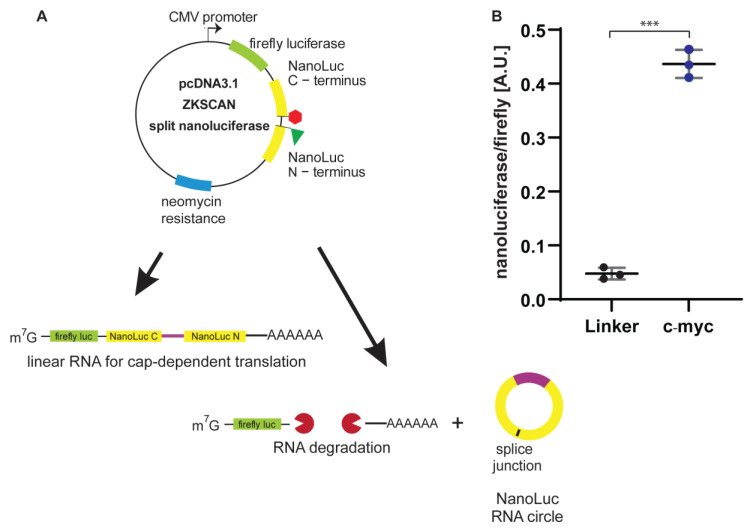
A split NanoLuc reporter plasmid with a firefly luciferase reporter as an internal control. (**A**) Schematic of the split NanoLuc reporter plasmid with the firefly luciferase inserted into the MCS of pcDN3.1. Upon transcription, the linear RNA contains both the firefly luciferase open reading sequence and the split NanoLuc ORF. The linear RNA yields active firefly luciferase protein, while the split NanoLuc protein remains inactive. Upon backsplicing, the NanoLuc ORF remains in the circular RNA, is fused and yields NanoLuc protein. (**B**) NanoLuc over firefly luciferase values is plotted for the linker control and the c-Myc cellular IRES. Experiments were performed in triplicate. Error bars represent the standard deviation. Significances were calculated using an unpaired *t* test with Welch’s correction with *p* ≤ 0.001 = ***.

To eliminate the need for co-transfection of the firefly luciferase report and improve ease of use, we next generated a reporter that encoded both firefly and NanoLuc proteins on the same mRNA (Figure 3A). We PCR-amplified the firefly ORF and inserted it into the NheI and BamHI sites of the linker or c-myc containing ZKSCAN split NanoLuc plasmids. Following confirmation by Sanger sequencing, these dual expression plasmids were separately transfected into HeLa cells, and firefly and NanoLuc expression were measured 24 h post-transfection. The nano to firefly ratio for the dual-luciferase linker construct was 0.048 ± 0.009 and was close to the average of 0.047 ± 0.012 measured during co-transfection of the ZKSCAN split-linker NanoLuc and pGL4.13 plasmids (Figure 1B). The average for the dual-luciferase c-Myc reporter was 0.437 ± 0.021, while the average for the co-transfected plasmids was 7.607 ± 0.505 (Figure 3B). Curiously, the observed difference between the dual-luciferase c-Myc and linker constructs was only approximately ninefold, while the difference from the co-transfection experiment was 161-fold. While the dual-luciferase plasmid generates one transcript that could either result in firefly or in NanoLuc expression, during the co-transfection experiments the firefly- and NanoLuc-encoding plasmids are independently transcribed, processed and translated, which could explain the observed differences in the ratio for the two approaches. 

## 4. Discussion

In this study, we replaced the split GFP within the pcDNA3.1(+) ZKSCAN1 MCS-WT Split GFP plasmid with a split NanoLuc gene to create a quantitative and sensitive reporter for circular RNA translation. We showed that an IRES and backsplicing are required to produce NanoLuc. We further tested the translation of putative IRES sequences from cellular sequences and ZIKV. Lastly, we inserted a firefly luciferase ORF upstream of the split nanoluciferase gene. This dual-luciferase construct no longer requires co-transfection of two plasmids, as cap-dependent and cap-independent reads can be measured from the same plasmid.

To overcome limitations of the traditional dicistronic luciferase assay, circular RNAs have been proposed to accurately measure IRES-mediated translation [22] with cellular backsplicing as a potential mechanism to generate these RNAs [24,25]. Here, we used a previously published plasmid containing intronic sequences from the human ZKSCAN1 gene that facilitate backsplicing to fuse a GFP ORF and translate GFP [27]. We adapted this plasmid to generate a quantitative reporter using a split NanoLuc gene. We showed that the production of NanoLuc requires splicing and a functional IRES (Figure 1B). Insertion of a short 9 bp unstructured linker showed higher levels of NanoLuc than the co-expression of N-terminal and C-terminal fragments from two co-transfected plasmids, suggesting that the inserted linker may allow for an RNA secondary structure formation that could function as a weak IRES. Since circular RNA molecules are more restricted in their RNA secondary structure formation, it may be challenging to take that into account when using RNA structure prediction algorithms. Alternatively, the linker sequence CCTTACTTC may contain a weak signal for N6-methyladenosine (m6A) modification and m6A modifications have been shown to facilitate the translation of circular RNAs [40]. Curiously, the insertion of the EMCV IRES in the reverse orientation (Figure 1B), despite forming hairpin stem-loops, did not allow for NanoLuc production. Together, these results suggest that stem-loop formation may be required however is not a sufficient characteristic to mediate translation from a circular RNA. Indeed, work by Weingarten-Gabbay et al. showed that not only RNA secondary structures but also base pairing with the 18S ribosomal RNA (rRNA) and other short sequence motifs can mediate cap-independent translation [41]. Such an interaction between helix 26 of the 18S rRNA and the hepatitis C virus (HCV) IRES domain IIId loop has been shown to be critical for HCV IRES function [42]. 

We further showed that, upon insertion of putative cellular IRES, NanoLuc activity could be measured. The c-Myc mRNA is known to be translated cap-independently, and extensive testing had been performed [34,35,43]. Similarly, evidence supporting the IRES within DAP5 is strong, as during the induction of apoptosis eIF4GI disappears while DAP5 is continuously produced [44]. In contrast, an IRES in c-Jun was reported in a few publications [36,37], although evidence for alternative cap-dependent translation mediated by eIF3d was described [45,46]. As expected, c-Myc and DAP5 IRESs were able to robustly facilitate NanoLuc translation. In contrast, translation from the c-Jun UTR was significantly weaker (Figure 2B), albeit higher than background levels from the linker and co-expression of N- and C-terminal fragments (Figure 1B). Together, these results suggest that the c-Jun IRES may be considered a weaker IRES. While we did not observe major differences in backsplicing efficiency amongst our constructs via northern blotting against the circular RNA splice junction, with the notable exception of the splice donor and acceptor site deletions that did not show any circular RNA product (Figure S2), others have reported that various IRES elements can impact backsplicing [47]. Weak splice sites within the IRES could facilitate the removal of the IRES from the circular RNA, which could yield false-negative results. In contrast, the inclusion of the KSHV vFLIP IRES seemed to cause concatemers [47]. Northern blotting using an antisense oligonucleotide against a NanoLuc portion shared between the linear and circular construct did not reveal any additional bands, suggesting that our constructs may not suffer from such artifacts. However, these observations indicate that additional experiments are crucial to correctly interpreting the translation efficiency of IRESs tested with a circular RNA reporter.

One feature of viral and cellular IRESs is that their translation continues during induction of the intergenic stress response and phosphorylation of eIF2α [48]. Similarly, treatment with sodium arsenite, tunicamycin and thapsigargin inhibited cap-dependent translation, while the EMCV IRES remained active and continued to translate NanoLuc protein. As a result, the ratio of cap-independent NanoLuc and cap-dependent firefly luciferase increased in response to chemical stressors (Figure 1C). 

We additionally used the split NanoLuc reporter to test another putative IRES, the Zika virus IRES. It had been reported that the Dengue virus could be translated cap-independently, although it was initially proposed that this mechanism was not IRES-mediated [38]. Several screens for proteins required by Dengue virus and Zika viruses revealed that they require ribosomal protein S25 (eS25) [49,50,51]. Interestingly, eS25 was shown to be important for IRES-mediated translation [52,53], questioning whether Dengue and Zika viruses might use an IRES for translation initiation. Song et al. tested whether Dengue and Zika viruses may utilize an IRES using a dual-luciferase reporter construct, which had a hairpin inserted upstream of the first cistron to prevent cap-dependent translation [39]. By expressing the human rhinovirus 2A protease in HEK 293T cells, the Dengue virus’ IRES remained active, which further validated the presence of an IRES in the Dengue and Zika virus [54]. Our split NanoLuc assay independently verified that the 5′ UTR but not the 3′ UTR possesses IRES activity (Figure 3B). Interestingly, the direct fusion of the 3′ and 5′ UTRs slightly reduced IRES activity, suggesting that certain flexibility is required to form the correct RNA secondary structure. By fusion with an upstream sequence as in a circular RNA construct, the flexibility to form RNA secondary structures may be constrained, which could reduce IRES activity.

For our initial co-transfection experiments, we employed two constructs, each with a different promoter (backsplice = CMV, pGL4.13 = SV40) to avoid promoter competition. However, we did notice variability in the data, likely due to unavoidable transfection efficiency. We generated and tested the transfection of a dual-luciferase version of the reporter to reduce variability. In this construct, the expression of NanoLuc and firefly luciferase is mutually exclusive with the linear transcript responsible for firefly luciferase expression, while the NanoLuc is only expressed upon backsplicing. Interestingly, the insertion of the firefly luciferase upstream reduced NanoLuc production in the plasmid containing the c-myc IRES, suggesting that insertion of an upstream sequence could influence the backsplicing rate and hence NanoLuc levels. Although our construct is the first utilizing luciferase enzymes, a similar construct encoding a Ruby Red fluorescent protein to measure cap-dependent translation and the split GFP to measure circular RNA translation has been used to perform a screen for putative IRESs [55]. From 55,000 oligonucleotides approximately 200 nucleotides in length, Chen et al. identified more than 17,000 sequences as putative circular RNA IRESs. Structured RNA elements and 18S rRNA complementarity were identified by Chen et al. as drivers for circular RNA translation. This finding is in agreement with the IRES screen performed by Weingarten-Gabbay [41], although the overall IRES activities observed with the two studies were low. Additionally, the overall number of identified IRES sequences by Chen et al. (~17,000) [55] is far greater than the number of previously expected IRESs [17]. This finding warrants additional research to determine if these putative IRESs are utilized in cells to express non-canonical peptides implicated in self-recognition by the immune system or have other functions [56,57]. The split NanoLuc dual-luciferase construct detailed herein, and the associated sensitive quantitative luminescence assay will be a useful tool to investigate these putative cellular IRESs in the future.

## 5. Conclusions

Circular RNAs have advantages over the dicistronic luciferase assay to measure IRES-mediated translation. Here, we adopted a previously published ZKSCAN split GFP to create a split NanoLuc construct. This split NanoLuc construct is another tool to help investigate circular RNA IRESs further and measure and compare their ability to mediate translation using a sensitive luminescence assay.

## Data Availability

Not applicable.

## Supplementary Materials

The following supporting information can be downloaded at: https://www.mdpi.com/article/10.3390/genes13020357/s1, Figure S1: gene block sequences; Figure S2: northern blot; Table S1: primer sequences.
