# Supplementary material for: A Split NanoLuc Reporter Quantitatively Measures Circular RNA IRES Translation
Source: Genes (Basel). 2022 Feb 16;13(2):357. doi: 10.3390/genes13020357 (PMC8871761; doi:10.3390/genes13020357)
Supplement: Supplementary file 1 [file genes-13-00357-s001.zip › genes-1576204-SI.pdf]

EMCV split nanoLuc 117/118

CGTACTAATGACTTTTTTTTTATACTTCAGGGCATCGCCGTGTTCGACGGCAAAAAGATCACTGTAACAGGGACCCT
GTGGAACGGCAACAAAATTATCGACGAGCGCCTGATCAACCCCGACGGCTCCCTGCTGTTCCGAGTAACCATCAAC
GGAGTGACCGGCTGGCGGCTGTGCGAACGCATTCTGGCGTAACCTGCAGGCGCCCCTCTCCCTCCCCCCCCCCTAA
CGTTACTGGCCGAAGCCGCTTGGAATAAGGCCGGTGTGCGTTTGTCTATATGTTATTTTCCACCATATTGCCGTCTT
TTGGCAATGTGAGGGCCCGGAAACCTGGCCCTGTCTTCTTGACGAGCATTCCTAGGGGTCTTTCCCCTCTCGCCAA
AGGAATGCAAGGTCTGTTGAATGTCGTGAAGGAAGCAGTTCCTCTGGAAGCTTCTTGAAGACAAACAACGTCTGT
AGCGACCCTTTGCAGGCAGCGGAACCCCCCACCTGGCGACAGGTGCCTCTGCGGCCAAAAGCCACGTGTATAAGA
TACACCTGCAAAGGCGGCACAACCCCAGTGCCACGTTGTGAGTTGGATAGTTGTGGAAAGAGTCAAATGGCTCTC
CTCAAGCGTATTCAACAAGGGGCTGAAGGATGCCCAGAAGGTACCCCATTGTATGGGATCTGATCTGGGGCCTCG
GTGCACATGCTTTACATGTGTTTAGTCGAGGTTAAAAAAACGTCTAGGCCCCCCGAACCACGGGGACGTGGTTTTC
CTTTGAAAAACACGATGATAATATGGCGTTAACCACCatggccGATATCATGGTCTTCACACTCGAAGATTTCGTTGG
GGACTGGCGACAGACAGCCGGCTACAACCTGGACCAAGTCCTTGAACAGGGAGGTGTGTCCAGTTTGTTTCAGAA
TCTCGGGGTGTCCGTAACTCCGATCCAAAGGATTGTCCTGAGCGGTGAAAATGGGCTGAAGATCGACATCCATGT
CATCATCCCGTATGAAGGTCTGAGCGGCGACCAAATGGGCCAGATCGAAAAAATTTTTAAGGTGGTGTACCCTGT
GGATGATCATCACTTTAAGGTGATCCTGCACTATGGCACACTGGTAATCGACGGGGTTACGCCGAACATGATCGA
CTATTTCGGACGGCCGTATGAGGTAAGAAGCAAGGTTTCATTTAGGGGAAGGG

Magenta and cyan: sequences overlapping with plasmid for Gibson Assembly cloning
EMCV IRES
ATG and TAA: Start and Stop codons for the nanoLuc open reading frame
CCTGCAGG and GATATC: SbfI and EcoRV cloning sites

Uganda ZIKV full 3-5 UTR split Minigenome insert 607nt

GCGAACGCATTCTGGCGTAACCTGCAGGgcaccaattttagtgttgtcaggcctgctagtcagccacagtttggggaaagctgtgcagcc
tgtaacccccccaggagaagctgggaaaccaagctcatagtcaggccgagaacgccatggcacggaagaagccatgctgcctgtgagcccctcaga
ggacactgagtcaaaaaaccccacgcgcttggaagcgcaggatgggaaaagaaggtggcgaccttccccacccttcaatctggggcctgaactgga
gactagctgtgaatctccagcagagggactagtggttagaggagacccccggaaaacgcaaaacagcatattgacgctgggaaagaccagagact
ccatgagtttccaccacgctggccgccaggcacagatcgccgaacttcggcggccggtgtggggaaatccatggtttctagttgttgatctgtgtgagtc
agactgcgacagttcgagtctgaagcgagagctaacaacagtatcaacaggtttaatttggatttggaaacgagagtttctggtcatgaaaaacccaa
agaagaaatccggaggattccggattgtcaatatgctaaaacgcggaGATATCATGGTCTTCACACTCGAAG

Uganda ZIKV full 5-3 UTR split Minigenome 1190 nt $209/159

GCGAACGCATTCTGGCGTAACCTGCAGGagttgttgatctgtgtgagtcagactgcgacagttcgagtctgaagcgagagctaacaaca
gtatcaacaggtttaatttggatttggaaacgagagtttctggtcgcaccaattttagtgttgtcaggcctgctagtcagccacagtttggggaaagctg
tgcagcctgtaacccccccaggagaagctgggaaaccaagctcatagtcaggccgagaacgccatggcacggaagaagccatgctgcctgtgagcc
cctcagaggacactgagtcaaaaaaccccacgcgcttggaagcgcaggatgggaaaagaaggtggcgaccttccccacccttcaatctggggcctg
aactggagactagctgtgaatctccagcagagggactagtggttagaggagacccccggaaaacgcaaaacagcatattgacgctgggaaagacc
agagactccatgagtttccaccacgctggccgccaggcacagatcgccgaacttcggcggccggtgtggggaaatccatggtttctatgaaaaaccca
aagaagaaatccggaggattccggattgtcaatatgctaaaacgcggaGATATCATGGTCTTCACACTCGAAG

ATG and TAA: Start and Stop codons
5′ UTR and 3′UTR sequences and the capsid protein open reading frame portion from the ZIKV genome
CCTGCAGG and GATATC: SbfI and EcoRV cloning sites

Figure S1: gene block sequences

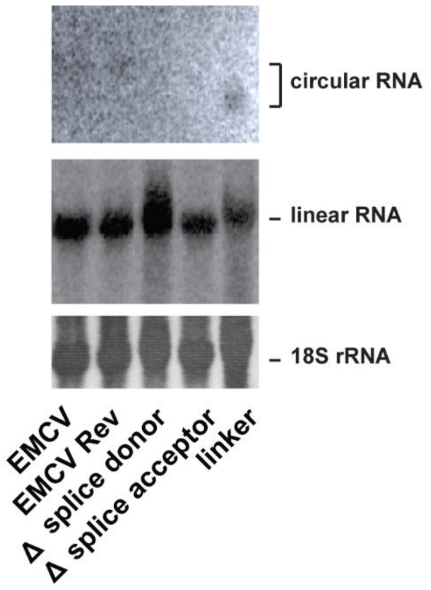

Figure S2: Northern blot analysis to detect circular, linear NanoLuc and 18S rRNA as a control. 10 µg of total RNA from stably transfected cells were separated by denaturing agarose gel electrophoresis, followed by northern blot analysis using an oligonucleotide hybridized to the NanoLuc splice junction to detect the circular RNA, and to the C-terminal fragment of the NanoLuc to detect the linear RNA.

Figure S2: northern blot

Table S1: primer sequences.

Primer Name	Primer Sequence	Purpose
EMCV Rev SbfI Rev	GGCCCTGCAGGggccatGGTGGTTAACGCCATATTATC	cloning
EMCV Rev EcoRV Fwd	GCGGATATCCGCCCCTCTCCCTCCCCCCCCCCT	cloning
SbfI-9 bp-EcoRV Fwd	GGCCTTACTTCGAT	cloning
SbfI-9 bp-EcoRV Rev	ATCGAAGTAAGGCCTGCA	cloning
nanoLuc N HindIII Fwd	GGGAAGCTTACCATGGTCTTCACACTCGAAGATTTC	cloning
nanoLuc N BamHI Rev	GCGGGATCCTTACTCATACGGCCGTCCGAAATAG	cloning
nanoLuc C HindIII Fwd	GGGAAGCTTACCATGGGCATCGCCGTGTTCGACGG	cloning
nanoLuc C BamHI	GCGGGATCCTTACGCCAGAATGCGTTCGCAC	cloning
Splice donor Fwd	AAGAAGCAAGGTTTCATTTAGGGGAAG	cloning
Splice donor Rev	CTCATACGGCCGTCCGAAATAGTCG	cloning
Splice acceptor Fwd	GGCATCGCCGTGTTCGACGGC	mutagenesis
Splice acceptor Rev	GAAGTATAAAAAAAAAGTCATTAGTACG	mutagenesis
SbfI Myc 5'UTR Fwd	GCG CCTGCAGG GAC CCC CGA GCT GTG CTG C	cloning
EcoRV Myc 5'UTR Rev	GGGGATATCCATGGTCTGGTT TTCCACTACCCGAAAAA	cloning
SbfI DAP5 5'UTR Fwd	GCG CCTGCAGG gccagcagtgagtcggagct	cloning
EcoRV DAP5 5'UTR Rev	GGG GATATC cgcactctccactttggcgg	cloning
SbfI cJun 5'UTR Fwd	GCG CCTGCAGGgctcagagttgcactgagtgtg	cloning
EcoRV cJun 5'UTR Rev	GGG GATATCcatagaacagtccgtcacttcacgtg	cloning
ZIKV SbfI 3UTR Fwd	GCGTAACCTGCAGGgcaccaattttagtgttgtcaggc	ZIKV insertion
ZIKV SbfI 5UTR Fwd	GCGTAACCTGCAGGagttgttgatctgtgtgagtcagact	ZIKV insertion
ZIKV SbfI Capsid Rev	CCATGATATCtccgcgttttagcatattgacaat	ZIKV insertion
ZSCAN Fwd	GTAAGAAGCAAGGTTTCATTTAGGG	sequencing, gene block amplification
ZSCAN Rev	CTGAAGTATAAAAAAAAAGTCATTAGTAC	sequencing, gene block amplification
nanoLuc Fwd	GGGGATATCATGGTCTTCACACTCGAAG	sequencing
nanoLuc Rev	GCGCCTGCAGGTTACGCCAGAATGCGTTCGC	sequencing and northern
nanoLuc junction	GCGATGCCCTCATACGGC	northern
NheI-FF Fwd	GGGGCTAGCACCATGGAAGACGCCAAAAACATAAAG	cloning
BamHI FF Rev	GCGGGATCCTTACACGGCGATCTTTCCGCCC	cloning
